# Characterization and Phylogenetic Analysis of the Mitochondrial Genome of *Glarea lozoyensis* Indicates High Diversity within the Order Helotiales

**DOI:** 10.1371/journal.pone.0074792

**Published:** 2013-09-25

**Authors:** Loubna Youssar, Björn Andreas Grüning, Stefan Günther, Wolfgang Hüttel

**Affiliations:** 1 Pharmaceutical Bioinformatics, Institute of Pharmaceutical Sciences; University of Freiburg, Freiburg, Germany; 2 Pharmaceutical and Medicinal Chemistry, Institute of Pharmaceutical Sciences, University of Freiburg, Freiburg, Germany; University of Lausanne, Switzerland

## Abstract

**Background:**

*Glarea lozoyensis* is a filamentous fungus used for the industrial production of non-ribosomal peptide pneumocandin B_0_. In the scope of a whole genome sequencing the complete mitochondrial genome of the fungus has been assembled and annotated. It is the first one of the large polyphyletic Helotiaceae family. A phylogenetic analysis was performed based on conserved proteins of the oxidative phosphorylation system in mitochondrial genomes.

**Results:**

The total size of the mitochondrial genome is 45,038 bp. It contains the expected 14 genes coding for proteins related to oxidative phosphorylation,two rRNA genes, six hypothetical proteins, three intronic genes of which two are homing endonucleases and a ribosomal protein rps3. Additionally there is a set of 33 tRNA genes. All genes are located on the same strand. Phylogenetic analyses based on concatenated mitochondrial protein sequences confirmed that *G. lozoyensis* belongs to the order of Helotiales and that it is most closely related to *Phialocephala subalpina*. However, a comparison with the three other mitochondrial genomes known from Helotialean species revealed remarkable differences in size, gene content and sequence. Moreover, it was found that the gene order found in *P. subalpina* and *Sclerotinia sclerotiorum* is not conserved in *G. lozoyensis.*

**Conclusion:**

The arrangement of genes and other differences found between the mitochondrial genome of *G. lozoyensis* and those of other Helotiales indicates a broad genetic diversity within this large order. Further mitochondrial genomes are required in order to determine whether there is a continuous transition between the different forms of mitochondri*a*l genomes or *G. lozoyensis* belongs to a distinct subgroup within Helotiales.

## Introduction

The filamentous fungus *Glarea lozoyensis* (ATCC 20868) was originally isolated by plating filtrates of pond water near Madrid, Spain [1]. It came to be known as a producer of pneumocandins, non-ribosomal peptides with strong inhibitory effect on fungal glucan biosynthesis. *G. lozoyensis* ATCC 74030 is a mutant strain of the wild type used for the production of the antimycotic drug Caspofungin from Pneumocandin B_0_ [2], [3]. The taxonomy of *G. lozoyensis* has been revised several times [4]. It was designated *Zalerion arboricola* first [1], however, after thorough analysis of morphological and molecular data, it was classified as a new anamorphic genus in the order Helotiales [4]. Through sequence analysis of the ITS region of a broad array of species in Helotiales the closest relatives of *G. lozoyensis* were narrowed to some *Cyathicula* De Not species (Helotiaceae) [5].

Since mitochondrial (mt) genomes often evolve faster than nuclear genomes [6], [7], [8], they have been successfully applied as markers in evolutionary biology [9], [10]. With the emergence of next-generation sequencing in the last years, the access to whole genomes has become easy and affordable. The number of completely sequenced mt genomes of filamentous fungi has increased dramatically [11], [12], so that it is possible to use them for phylogenetic studies [13], [14], [15], [16], [17], [18]. The Fungal Mitochondrial Genome Project was launched over a decade ago [19]. Now, more than 80 fungal mitogenomes are available. Nevertheless, only four of the Helotiales order, *Phialocephala subalpina*, *Sclerotinia sclerotiorum, Botrytis cinera* and *Marssonina brunnea* (incomplete annotation) have been sequenced so far (Table 1).

**Table 1:** Selected fungus species with published mt genomes.

Species	Class	Order	Length (bp)	Accession Nr
*Glarea lozoyensis*	Leotiomycetes	Helotiales	45,038	GenBank: KF169905
*Phialocephala subalpina*	Leotiomycetes	Helotiales	43,742	GenBank: NC_015789
*Botrytis cinerea*	Leotiomycetes	Helotiales	80,799	Broad Institute^1^
*Sclerotinia sclerotiorum*	Leotiomycetes	Helotiales	128,852	Broad Institute^2^
*Marssonina brunnea*	Leotiomycetes	Helotiales	70,379	Genbank: JN204424
*Gibberella zeae*	Sordariomycetes	Hypocreales	95,676	GenBank: NC_009493
*Gibberella miniliformis*	Sordariomycetes	Hypocreales	53,753	GenBank: NC_016687
*Hypocrea jecorina*	Sordariomycetes	Hypocreales	42,13	GenBank: NC_003388
*Beauveria bassiana*	Sordariomycetes	Hypocreales	29,961	GenBank: NC_010652
*Metarhizium anisopliae*	Sordariomycetes	Hypocreales	24,673	GenBank: NC_008068
*Aspergillus niger*	Eurotiomycetes	Eurotiales	31,103	GenBank: NC_007445
*Penicillium marneffei*	Eurotiomycetes	Eurotiales	35,438	GenBank: NC_005256
*Emericella nidulans*	Eurotiomycetes	Eurotiales	33,227	GenBank: NC_017896
*Aspergillus kawachi*	Eurotiomycetes	Eurotiales	31,222	GenBank: AP012272
*Arthroderma obtusum*	Eurotiomycetes	Onygenales	24,101	GenBank: NC_012830
*Trichophyton mentagrophytes*	Eurotiomycetes	Onygenales	24,297	GenBank: NC_012826
*Neurospora crassa*	Sordariomycetes	Sordariales	6,484	Broad Institute^3^
*Podospora anserina*	Sordariomycetes	Sordariales	94,192	GenBank: NC_001329
*Glomerella graminicola*	Sordariomycetes	Glomerellales	39,649	GenBank: CM001021
*Verticillium dahliae*	Sordariomycetes	Glomerellales	27,184	GenBank: NC_008248
*Phaeosphaeria nodorum*	Dothideomycetes	Pleosporales	49,761	GenBank: NC_009746
*Mycosphaerella graminicola*	Dothideomycetes	Capnodiales	43,964	GenBank: NC_010222
*Candida albicans*	Saccharomycetes	Saccharomycetales	4,042	GenBank: NC_002653
*Ogataea angusta*	Saccharomycetes	Saccharomycetales	41,719	GenBank: NC_014805
*Pichia pastoris*	Saccharomycetes	Saccharomycetales	35,683	GenBank: NC_015384

1 *Botrytis cinerea* Sequencing Project.

2 *Sclerotinia sclerotiorum* Sequencing Project.

3 *Neurospora crassa* Sequencing Project, (http://www.broadinstitute.org/).

A fungal mt genome typically contains 14 conserved protein-coding genes, 22–26 tRNA genes, and 2 rRNA genes arranged very likely in circular form [20], [21], [22], [23]. The mtDNA divergence between different fungal species is characterized by variations in intergenic regions, intronic sequences, and in the order of genes [24], [25].

We have identified the mitochondrial genome of *G. lozoyensis* ATCC 74030 within the whole genome of the fungus, which was assembled by combination of data from Illumina MP and PE sequencing [26]. As there are only very few mt genomes known from species of the large Helotiales order, we decided to annotate this genome in detail and to investigate its relation to the known mt genomes of Helotiales. These were also used as main reference for a thorough manual revision of the automatic annotation obtained from diverse bioinformatic tools.

## Materials and Methods

### Cultivation and DNA Preparation

*G*. *lozoyensis* ATCC 74030 was grown for 20 days at 25°C on a YM agar plates (yeast extract 0.1%, malt extract 1.0%, agar 1.5% in H_2_O). The mycelium was scraped off the plate, freeze-dried and ground to a fine powder. Total DNA was isolated with a DNeasy Plant Mini Kit (Qiagen) according to the manufacturer’s instructions.

### Sequencing and Assembly

Whole genome shotgun sequencing of *G. lozoyensis* ATCC 74030 was performed by sequencing a paired-end library and an additional mate-pair library with an Illumina HiSeq 2000 sequencer. About 38 Gbp of reads with an average length of 75 bp were assembled to contigs using the CLC Genomics Workbench (CLCbio). Scaffold N50 is estimated to 870,933. A single 45,038 bp scaffold representing the complete mtDNA was identified by sequence similarity search to known fungal mt genomes.

### Mitochondrial Genome Annotation

Potential open reading frames (ORFs) in the mtDNA sequence of *G. lozoyensis* were identified using Prodigal [27] and ORF Finder based on genetic code 4 (www.ncbi.nlm.nih.gov/gorf/gorf.html) performed with a Galaxy public server as a platform [28], [29]. Functional annotation was performed with BLASTp [30], BLASTx [30], Pfam [31] and MFannot (http://megasun.bch.umontreal.ca/cgi-bin/mfannot/mfannotInterface.pl). The mitochondrial genomes of *P. subalpina* [GenBank: JN031566] and *S. sclerotiorum* (http://www.broadinstitute.org) were used as references for manual annotation. tRNA genes were predicted by tRNAscan-SE [32], ARAGORN [33], RNAWEASEL [34], [35] and ARWIN [36] using the default settings for mt genomes. A potential tRNA was considered as proven when it was found by at least two of these tools. Ribosomal RNA genes were identified by sequence similarity with the corresponding genes of *P. subalpina* and *S. sclerotiorum*. Introns were predicted manually using BLASTx [30].

### Phylogenetic Analysis

To determine the evolutionary background of *G. lozoyensis,* a concatenation of 12 OXPHOS genes (atp6, cob, cox1, cox2, cox3, nad1, nad2, nad3, nad4, nad4L, nad5 and nad6) was compared with analogous sets of genes from 24 mt genomes published in GenBank. Protein sequence alignment was carried out for each protein using ClustalW with default options through a Galaxy server [37], [28], [29]. The aligned protein sequences were used to construct a maximum likelihood tree with PhyML 3.0 using LG as evolutionary model [38]. The reliability for internal branch was assessed using the aLRT test (SH-Like) as recommended in PhyML. The graphic representation was performed with Treeview (http://taxonomy.zoology.gla.ac.uk/rod/rod.html) and manual editing.

### Genbank Accession Number

The *G. lozoyensis* ATCC 74030 mt genome sequence is deposited in GenBank under accession number KF169905.

## Results and Discussion

### General Features

**Figure 1:**
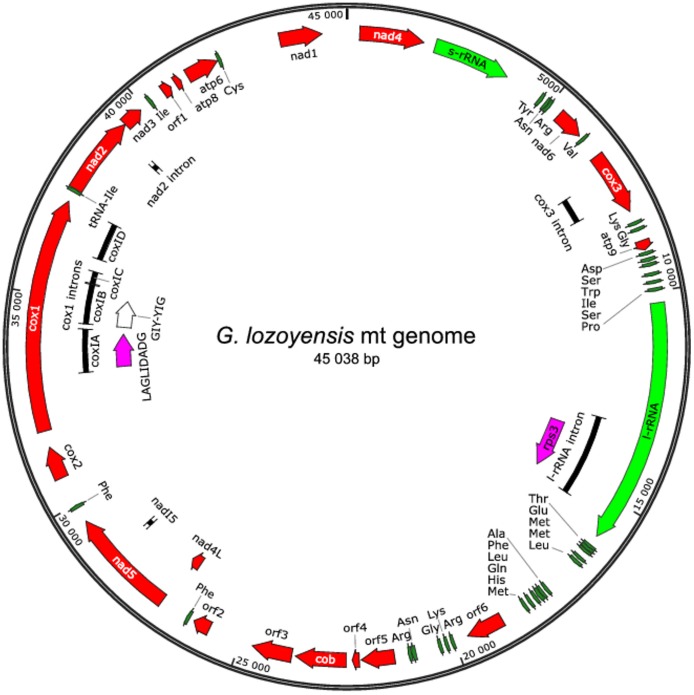
Circular mapping of the complete mt genome of *G. lozoyensis*. All genes are located on the plus-strand. Protein coding genes (red; atp: ATPase synthase subunits, cob: cytochrome b, cox: cytochrome oxidase subunit, nad: NADH dehydrogenase subunit, orf: hypothetical proteins); ribosomal subunits (green; rns: small rRNA, rnl: large rRNA); tRNA genes (dark green); introns (black) and intronic proteins (purple, if protein coding). The precise positions of genes and introns are listed in table S1.

The mitochondrial genome of *G. lozoyensis* ATCC 74030 comprises 45,038 bp, which is in the same range as several other mt genomes in Pezizomycotina (Table 1). 31.8% is covered with intergenic spacers of 3–1131 bp length and 15.04% with seven introns (Table 2). Among the five mt genomes reported, three are relatively large (Table 1) and only that of *P. subalpina* (43.7 kbp) is similar to that of *G. lozoyensis* (Table 1). The differences in genome size are marked by a multitude of introns and endonucleases in *B. cinerea* and *S. sclerotiorum* and a large intergenic region in *M. brunnea*. With 58.1% of the genome encoding structural genes, the mt genome of *G. lozoyensis* is rather compact (Table 2). It encodes the large and the small ribosomal RNA subunit (rnl and rns), 33 tRNAs, 14 putative proteins of the oxidative phosphorylation system (OXPHOS), 6 hypothetical proteins and 3 intronic proteins, of which one is ribosomal protein RPS3 and two are homing endonucleases (HE) (Figure 1, Table 2). As in mitochondria of most other ascomycetes, all genes and tRNAs are found on the plus strand [13], [14], [15], [16], [17], [18]. The overall G+C content of the mt genome is 29.8% (Table 2), consistent with the characteristic AT-rich nature of fungal mt genomes. The G+C content of regions encoding RNA genes is usually higher than the genome wide average. In *G. lozoyensis* it is 31% (Table 2), which is similar to other fungal mitochondria [39].

**Table 2:** General features of the mt genome of *G. lozoyensis.*

Genomes features	Value
Genome size (bp)	45,038
G+C content (%)	29.8
No. of protein-coding genes	20
G+C content of protein-coding genes (%)	31.0
Structural proteins coding exons (%)	38.7
No. of rRNAs/tRNAs	2/33
G+C content of RNA genes (%)	35.9
rRNAs+tRNAs (%)	14.4
Coding regions (%)	58.1
Intergenic regions (%)	31.8
No. of introns	7
No. of intronic ORFs	3 + (1)*
Introns (%)	15.0

* Eroded protein.

The mt genome of *G. lozoyensis* was compared with the fully annotated mt genomes of the Helotiales order, i.e., those of *P. subalpina* and *S. sclerotiorum*. BLASTn alignment [40] gave a sequence identity of 27% with the mt genome of *P. subalpina* and 19% with that of *S. sclerotiorum*. Moreover, DNA dot plot analysis showed that the mt genome of *G. lozoyensis* is colinear with that of *P. subalpina* (Figure S1) [40]. Considering also the similar size, the mt genome of *P. subalpina* was chosen as the main reference for the manual annotation of the mt genome of *G. lozoyensis*.

### Protein Coding Genes

The following genes encoding proteins involved in respiratory chain complexes (OXPHOS) were found in the *G. lozoyensis* mt genome: atp6, atp8, atp9, cox1-3, cob, nad1-6 and nad4L (Figure 1, Table S1). Moreover, there are three intronic proteins, of which one is eroded (see section introns), and six hypothetical proteins (ORF1-6). All 14 OXPHOS proteins are highly conserved in *P. subalpina* (Table S1). Differences in sequence mainly refer to intronic proteins, which were not found in *P. subalpina* [13]. For instance, the large ribosomal subunits (rnl) in *G. lozoyensis* shares only a sequence identity of 45% with the corresponding feature in *P. subalpina.* This is mainly due to the presence of intron IE (2250 bp) in *G. lozoyensis* rnl, which also includes the gene for ribosomal protein S3 (rps3). In *P. subalpina* IE does not exist and rps3 forms a separate ORF [13]. ORF included in introns are presumably an excellent criterion for inferring phylogenetic relationships of fungi. In Pezizomycotina, the intronic rps-like protein may even play a role in maintaining the integrity of the mt genome [41].

The length of the cox1 gene varies widely within the investigated species. In *G. lozoyensis* it includes 5,412 bp, in *S. sclerotiorum* 12,458 bp and in *P. subalpina* only 1,720 bp. In contrast to the latter, *S. sclerotiorum* and *G. lozoyensis* cox1 contain several intronic proteins such as GIY-YIG and LAGLIDADG. For that reason, *S. sclerotiorum* cox1 was chosen as additional reference for intronic gene annotation in *G. lozoyensis* cox1. The *G. lozoyensis* GIY-YIG (842 bp) shares 87% sequence identity with a GIY-YIG of *S. sclerotiorum* (SS1G_20030.1; 816 bp). Since it is an eroded ORF, the DNA sequence was used for comparison instead of the protein sequence. The LAGLIDADG gene of *G. lozoyensis* shows only 17% identity to a LAGLIDADG from *S. sclerotiorum* (SS1G_20022; 323 aa, Broad Institute). However, a BLASTp search on NCBI database nr resulted a maximum identity of 76% with a LAGLIDADG of the Pezizomycotum *Ajellomyces dermatitidis* SLH14081. Of the six hypothetical proteins (ORF1-6) only ORF2 and ORF6 share similarities with known sequences. A part of ORF2 (132 aa) is similar to cytochrome c oxidase subunit I (cox1) of *G. lozoyensis*, even though only 39% of ORF2 is covered (e-value 3e^−15^). The sequence identity between both is 78% at the amino acid level. The occurrence of this fragment may be explained by partial gene duplication due to the presence of a transposon. In general, the evolution of gene orders in Pezizomycotina is mainly characterized by transpositions [13]. For example, in eight mt genomes of *Phialocephala* species, a duplication of the region around atp9 was found [13]. However, to confirm the transposon hypothesis in *G. lozoyensis*, more mt genomes of the same genus are required. ORF6 was found to be similar with a putative protein (ORF2) in the mt genome of *P. subalpina* (e-value: 7e^−39^, 46% identity and 90% coverage). No significant sequence similarities (BLASTp) or conserved domains (InterProScan [42]) were found for the other putative proteins.

### Introns

A total of eight introns were identified in the coding genes of the *G. lozoyensis* mt genome by BLASTx search and sequence alignment (nr-database) [30]. Four of them are located in cox1 (coxIA, coxIB, coxIC and coxID). The others were found in the large ribosomal subunit rnl (intron IE), cox3, nad2, and nad5 (Figure 1, Table S1). coxIA and IE belong to introns group I, which is dominating in fungal mt genes, while in plant mt genes group II introns are found more frequently [43]. It is characteristic for group I introns is that all upstream exons end with a “T” and all introns end with a “G”. The conserved stems [44] were found in both genes (data not shown). Group I introns are considered to be mobile genetic elements interrupting protein-coding and structural RNA genes [45]. Most of them carry a “homing endonuclease gene” (heg) encoding a DNA endonuclease (HE), which catalyzes in the transfer and site-specific integration (“homing”) of the intron [46], [47], [48]. There are four families of DNA endonucleases (HEs) [49] denoted by the presence of conserved amino acid sequence motifs: GIY-YIG, HC-box, HNH and LAGLIDADG [50], [51]. Among these the LAGLIDADG endonucleases form the largest group. They are encountered in some bacteria and bacteriophages as well as in organelle genomes of protozoans, fungi, plants, and sometimes in early branching Metazoans [52]. Two forms can be distinguished: Proteins with a single LAGLIDADG motif which dimerize and double-motif forms derived form a gene fusion of two monomeric forms [53]. In the *G. lozoyensis* mt genome an intact ORF encoding a putatively functional HE referred to LAGLIDADG (342aa) was found in cox1-intron coxIA. A Pfam analysis resulted only one LAGLIDADG motive (Table S1), so that it can be considered as the single-motif form of LAGLIDADG. Moreover, in coxIB a frameshifted and inactive ( = “eroded”) heg known as GIY-YIG (835 bp) with several stop codons in the sequence was found. Despite being inactive, the conserved domain of GIY-YIG was still identified by Pfam (see section Protein coding genes and Table S1). Eroded hegs are characterized by several point and length mutations resulting in frameshifts and stop codons interrupting the ORF [54]. In species from the basal fungal lineages many introns in the long cox1-gene carry eroded hegs [55]. The erosion is generally regarded as a preliminary step before complete elimination of the intron [49]. The presence of intact and eroded hegs strengthens the hypothesis that numerous events of loss and gain have occurred during evolution. Besides the heg, coxID contains also a DUF3839 (PF12943) conserved domain, whose function is unknown. Furthermore, in the intron of the large ribosomal subunit (IE) we found a protein encoding for ribosomal protein S3 known as rps3 (519aa) (Table S1).

### Genetic Code and Codon Usage

The codon usage of the *G. lozoyensis* mitochondrial ORFs was analyzed using genetic code four, which is common for Pezizomycotina mtDNA [56]. Most protein-coding ORFs start with the orthodox translation initiation codon ATG. Exceptions are: nad2 and cox3 with GTG, cox2 with TTA and cox1 with TTG. Six genes end with the stop codon TAG: cob, orf3, orf2, nad3, atp6, and nad5; all others with TAA, which is the preferred termination codon for fungal mt genes [22], [57]. The intronic proteins, ribosomal protein S3 (rps3) and the putative LAGLIDADG endonuclease, start with ATG but the stop codon is TAA for rps3 and TAG for LAGLIDADG. The most commonly used amino acid in the 22 protein genes is leucine followed by isoleucine (Table 3). Similar results are reported for other fungi [14], [18]. As expected from the high AT content (79%), the most frequently used codons are composed exclusively of “U” and “A”, e.g. UUA (2.78%), AUA (1.88%), AAU (1.38%), UUU (1.82%), AAA (1,27%), UAU (1.32%) and AUU (1.20%) (Table 3). The codons UGC, CGC, CUC, CGA, CGG, UCG are underrepresented, being used one to twenty times less than the GC -rich codons (Table 3).

**Table 3:** Codon usage of protein-coding genes in *G. lozoyensis* mt genome.

Codon	AA	%	Codon	AA	%
GCA	A	0.52	CCA	P	0.28
GCC	A	0.28	CCC	P	0.18
GCG	A	0.12	CCG	P	0.10
GCU	A	0.83	CCU	P	0.73
UGC	C	0.03	CAA	Q	0.55
UGU	C	0.29	CAG	Q	0.20
GAC	D	0.21	AGA	R	0.65
GAU	D	0.86	AGG	R	0.15
GAA	E	0.78	CGA	R	0.08
GAG	E	0.30	CGC	R	0.03
UUC	F	0.56	CGG	R	0.06
UUU	F	1.82	CGU	R	0.14
GGA	G	0.37	AGC	S	0.22
GGC	G	0.11	AGU	S	0.95
GGG	G	0.28	UCA	S	0.47
GGU	G	1.06	UCC	S	0.18
CAC	H	0.23	UCG	S	0.07
CAU	H	0.40	UCU	S	0.83
AUA	I	1.88	ACA	T	0.63
AUC	I	0.23	ACC	T	0.22
AUU	I	1.20	ACG	T	0.11
AAA	K	1.27	ACU	T	0.73
AAG	K	0.39	GUA	V	0.77
CUA	L	0.49	GUC	V	0.12
CUC	L	0.07	GUG	V	0.31
CUG	L	0.14	GUU	V	0.85
CUU	L	0.59	UGG	W	0.12
UUA	L	2.78	UGA	W	0.34
UUG	L	0.44	UAC	Y	0.43
AUG	M	0.79	UAU	Y	1.32
AAC	N	0.51	UAA	Stop	0.18
AAU	N	1.38	UAG	Stop	0.16

The overall percentage of codon usage in the protein coding genes atp6, atp8, atp9, cob, cox1, cox2, cox3, nad1, nad2, nad3, nad4, nad4L, nad5, nad6, orf 1, orf 2, orf 3, orf 4, orf 5, orf 6, rps3, intron protein (IA), intron protein (IB) and intron protein (IE) is depicted.

### tRNA Genes

33 tRNA genes were identified in the *G. lozoyensis* mt genome, which is more than the 22–26 tRNA genes typically found in fungal mt genomes (Table 4, Figure 1) [20], [21], [22], [23], [32], [33], [36]. However, in Helotiales the numbers of tRNA genes appears to be generally higher, e.g. in *S. sclerotiorum* there are 33 tRNAs, in *B. cinera* and *M. brunnea* 31 tRNAs (predicted by TRNAscan tool [32]). In contrast, only 27 tRNAs were found in *P. subalpina*. Further mt genome sequences are required to confirm this tendency. The majority of *G. lozoyensis* mitochondrial tRNA genes are organized into two dense clusters. The set of 33 tRNA genes is sufficient to decode all codons in the predicted ORFs, lessening the need for tRNA import from the cytoplasm into the mitochondrium [58]. For lysine, asparagine and glycine two tRNA genes were found with the same anticodon. Furthermore, two tRNAs with different anticodons were identified for arginine, serine, phenylalanine and leucine. For methionine and isoleucine there are three tRNAs genes with identical anticodons.

**Table 4:** tRNAs in the mt genome of G. lozoyensis.

AA	Anticodon	AA	Anticodon
Asn	GUU*	Thr	UGU
Arg	UCG*	Glu	UUC
Arg	UCU	Leu	UAG
Val	UAC	Leu	UAA
Lys	UUU*	Ala	UGC
Gly	UCC*	Phe	AAA*
Asp	GUC	Phe	GAA
Ser	UGA	Gln	UUG
Ser	GCU	His	GUG
Trp	UCA	Met	CAU**
Ile	GAU**	Cys	GCA
Pro	UGG	Tyr	GUA
Ter	UUA*		

* Two tRNAs with the same anticodon.

** Three tRNAs with the same anticodon.

### Phylogeny and Comparative Genomics

**Figure 2:**
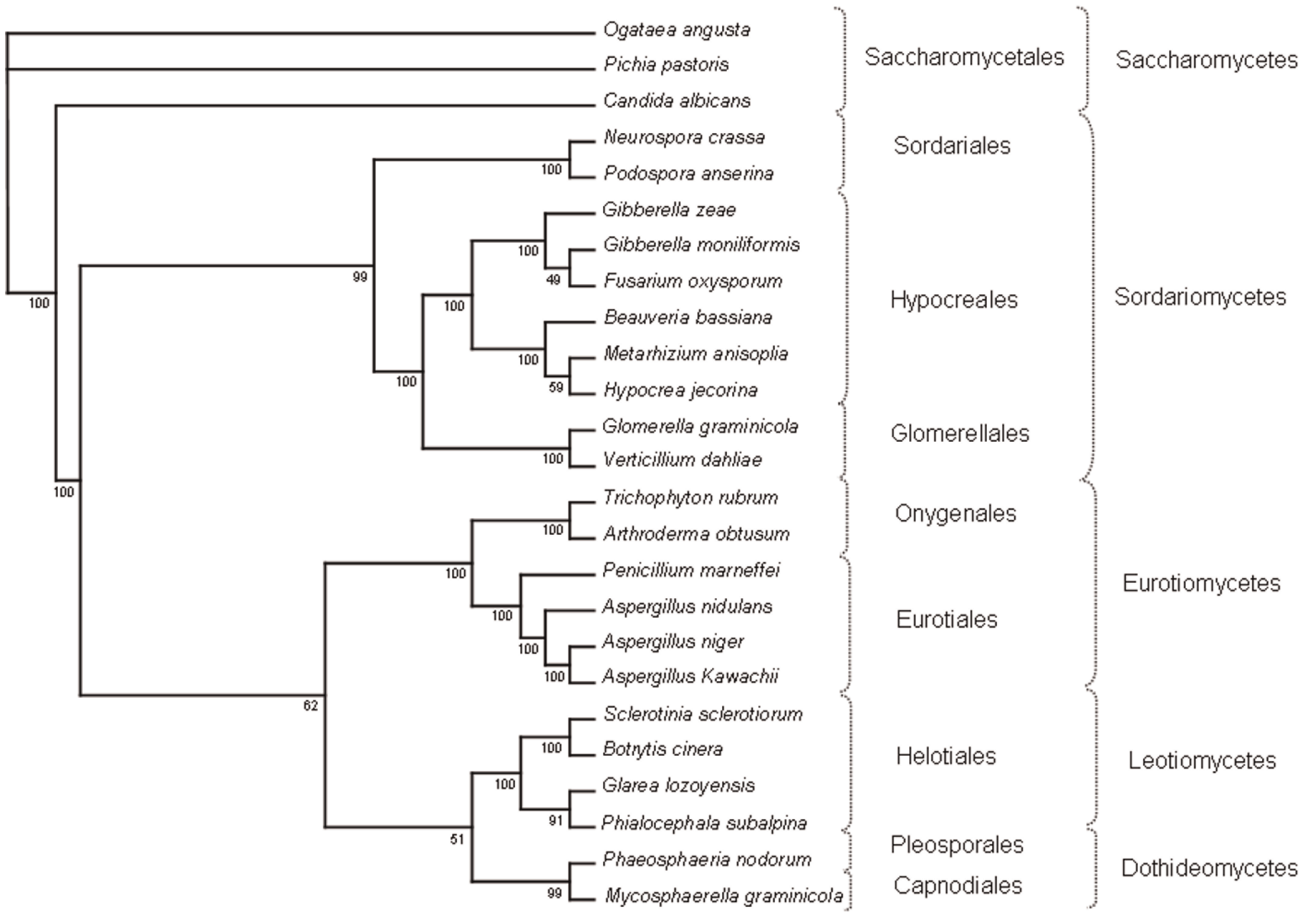
Phylogenetic analysis of *G. lozoyensis* based on mitochondrial protein sequences. The evolutionary history was inferred by using the Maximum Likelihood method in PhyML 3.0 [38]. The tree was based on 12 OXPHOS proteins ATP6, COB, COX1, COX2, COX3, NAD1, NAD2, NAD3, NAD4, NAD4L, NAD5 and NAD6 from 25 fungi: *G. zeae* (NC_009493), *G. moniliformis* (NC_016687), *F. oxysporum* (NC_017930), *H. jecorina* (NC_NC003388), *M. anisopliae* (NC_008068), *B. bassiana* (NC_010652), *G. graminicola* (CM001021), *V. dahliae* (NC_008248), *N. crassa* (NC_001570), *P. anserina* (NC_001329), *P. nodorum* (NC_009746), *M. graminicola* (NC_010222), *P. subalpina* (NC_015789), *T. rubrum* (NC_012824), *A. obtusum* (NC_012830), *P. marneffei* (NC_005256), *E. nidulans* (NC_017896), *A. niger* (NC_007445), *A. kawachi* (AP012272), *C. albicans* (NC_002653), *O. angusta* (NC_014805), *P. pastoris* (NC_015384), *S. sclerotiorum* and *B. cinera* (http://www.broadinstitute.org/).

**Figure 3:**
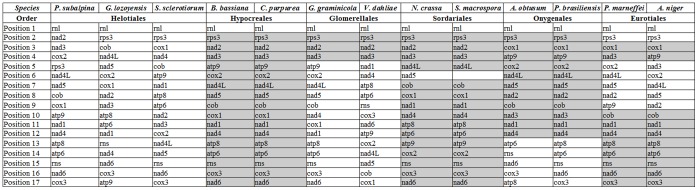
Mitochondrial gene order of 12 fungi species from 6 orders. The orders are: Helotiales, Hypocreales, Glomerellales, Sordariales, Onygenales and Eurotiales. The following genes were considered for gene order comparison: atp6, atp8, atp9, cob, cox1, cox2, cox3, nad1, nad2, nad3, nad4, nad4L, nad5, nad6 and rps3. Grey columns indicate coordination in genes arrangement for each order. rnl was defined arbitrarily as the first gene in each set. (*P*. *subalpina*: *Phialocephala subalpina*, *G*. *lozoyensis*: *Glarea lozoyensis*, *S*. *sclerotiorum*: *Sclerotinia sclerotiorum*, *B*. *bassiana*: *Beauveria bassiana*, *C*. *purpurea*: *Claviceps purpurea*, *G. graminicola*: *Glomerella graminicola*, *V*. *dahliae*: *Verticillium dahliae*, *N*. *crassa*: *Neurospora crassa*, *S. macrospora*: *Sordaria macrospora*, *A*. *obtusum*: *Arthroderma obtusum*, *P*. *brasiliensis*: *Paracoccidioides brasiliensis*, *P*. *marneffei*: *Penicillium marneffei*, *A*. *niger*: *Aspergillus niger.*

Since mt genomes often evolve faster than nuclear genomes, especially in intergenic regions [59], [60], mitochondrial markers were successfully applied in evolutionary biology [61], [62], [63]. In Pezizomycotina, completely sequenced and annotated mt genomes are available for members of Eurotiomycetes and Sordariomycetes [11], [12], [64]. But only three genomes with complete draft annotations are available for Helotialean species (Table 1). The Helotiales is one of the most diverse fungal order with more than 350 genera and over 2,000 species including many important plant pathogens [65]. In order to gain additional evidence for the classification of *G. lozoyensis*, we compared the amino acid sequences of 12 OXPHOS proteins (atp6, cox1-3, cob, nad1-6, nad4L) with those from 24 other fungi to build a phylogenetic tree (Figure 2). Most nodes in this tree have high bootstrap values, which indicate the robustness of the computed tree. Five classes of filamentous ascomycetes are clearly distinguished: Dothideomycetes; Leotiomycetes, Sordariomycetes, Eurotiomycetes and Saccharomycetes (Figure 2). As found already in other studies [44], the mt genomes of yeast species cluster apart from those of filamentous fungi. *G. lozoyensis* is found amongst other species of the Helotiales order with high bootstrap support. This placement is in line with previous observations based on nuclear ribosomal internal transcribed spacers (ITS) [8]. The closest relative of *G. lozoyensis* was found to be *P. subalpina*. This is consistent with the high similarities found already in BLASTp analyses (see above and Table S1). A close relation between protein sequence similarity and a uniform organization of mt genomes has been found for the orders Onygenales [39] and Sordariales [18]. The arrangement of mt genes might even be used as a reference to derive a common evolutionary route in fungi. We compared the mt gene order in *G. lozoyensis* with that of the Helotialean species with sequenced and annotated mt genome, i.e. *P. subalpina* and *S. sclerotiorum* (Figure 3). It was found that the gene organization in *G. lozoyensis* deviates significantly from both fungi (Figure 3). Already the mt genomes of *P. subalpina* and *S. sclerotiorum* differ considerably [15]. Anchored genome alignments for the two adjacent species *G. lozoyensis* and *P. subalpina* show no close genome rearrangements (Figure S2A). The similar result was obtained when *S. sclerotiorum* was included in the analysis (Figure S2B). In contrast, there is a complete synteny in gene order between *S. sclerotiorum* and *B. cinerea* [66]. Additional mt genomes of Helotiales species are required to allow further conclusions about the considerably diverse mt gene arrangements in this order.

## Conclusion

We have identified the complete mt genome of *G. lozoyensis* on a scaffold obtained by whole-genome sequencing. Previous studies based on RAPD, microsatellite-primed PCR and nuclear ribosomal internal transcribed spacers (ITS), suggest that *G. lozoyensis* belongs to the order Helotiales [5]. Our mt genome analysis clearly confirms that, BLASTp analysis of *G. lozoyensis* mt protein sequences yielded the corresponding genes from *P. subalpina* (Helotiales) as closest homologs (identities 70–100%); even though, not to all genes a homolog was found in *P. subalpina.* In a phylogenetic analysis based on the mt proteins of 24 fungi, *G. lozoyensis* was clearly classified as Helotiales with *P. subalpina* as closest relative. However, an anchored genome alignment of the Helotialean species *G. lozoyensis*, *P. subalpina* and *S. sclerotiorum* revealed that there is no synteny between these three apparently closely related species. This clearly demonstrates that species within the large Helotiales order species can be highly diverse. More mt genomes of the order Helotiales are required to find out whether the gaps between the differently ordered mt genomes can be fully closed or gene arrangements are in overall very diverse within this order.

## Supporting Information

Figure S1 ***G. lozoyensis*** **mt genome is colinear with that of** ***P. subalpina*** **.** Dotplot of mt genomes based on BLASTn analysis (http://blast.ncbi.nlm.nih.gov/Blast.cgi) with an e-value cutoff of 10^−10^. Sequence lengths are given along the axes in kbp. The shaded cells in the matrix indicate identical residues. a) *G. lozoyensis* and *P. subalpina*. b) *G. lozoyensis* and *S. sclerotiorum*.
(PDF)

Figure S2 **Mauve genome comparison.** Multiple alignments of four helotialean species, *G. lozoyensis*, *P. subalpina*, *S. sclerotiorum* and *B. cinerea* [67] were performed with the Mauve software package [67]. Locally collinear blocks (LCB) of the genome sequences are shown in identical colors and are connected with lines. For the genome of *G. lozoyensis* the annotation is displayed to allow the assignment of genes to LCBs. a) Alignment of *G. lozoyensis* and *P. subalpina*. b) Alignment of *G. lozoyensis*, *P. subalpina* and *S. sclerotiorum*.
(PDF)

Table S1 Mitochondrial genes annotation in *Glarea lozoyensis* and comparison to corresponding genes/proteins in *Phialocephala subalpine*.
(XLS)
