# Supplementary material for: Characterization and Phylogenetic Analysis of the Mitochondrial Genome of Glarea lozoyensis Indicates High Diversity within the Order Helotiales
Source: PLoS One. 2013 Sep 25;8(9):e74792. doi: 10.1371/journal.pone.0074792 (PMC3783487; doi:10.1371/journal.pone.0074792)
Supplement: Figure S1 — G. lozoyensis mt genome is colinear with that of P. subalpina . Dotplot of mt genomes based on BLASTn analysis (http://blast.ncbi.nlm.nih.gov/Blast.cgi) with an e-value cutoff of 10−10. Sequence lengths are given along the axes in kbp. The shaded cells in the matrix indicate identical residues. a) G. lozoyensis and P. subalpina. b) G. lozoyensis and S. sclerotiorum. (PDF) [file pone.0074792.s001.pdf]

A

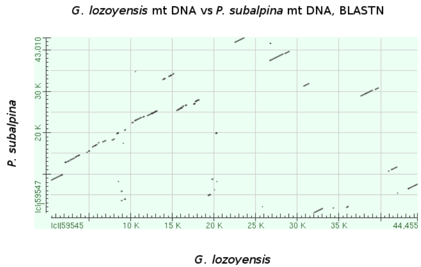
G. lozoyensis mt DNA vs P. subalpina mt DNA, BLASTN
P. subalpina
43,010
30 K
20 K
lcl|59547
lcl|59545
10 K
15 K
20 K
25 K
30 K
35 K
44,455
G. lozoyensis

B

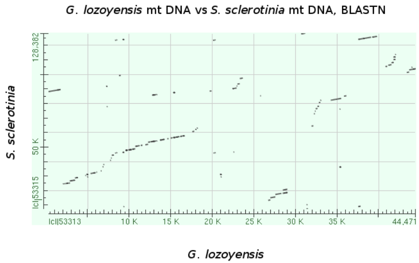
G. lozoyensis mt DNA vs S. sclerotinia mt DNA, BLASTN
S. sclerotinia
128,382
50 K
lcl|53315
lcl|53313
10 K
15 K
20 K
25 K
30 K
35 K
44,471
G. lozoyensis
