# Supplementary figures and images for: Characterization and Phylogenetic Analysis of the Mitochondrial Genome of Glarea lozoyensis Indicates High Diversity within the Order Helotiales
Source: PLoS One. 2013 Sep 25;8(9):e74792. doi: 10.1371/journal.pone.0074792 (PMC3783487; doi:10.1371/journal.pone.0074792)

**A**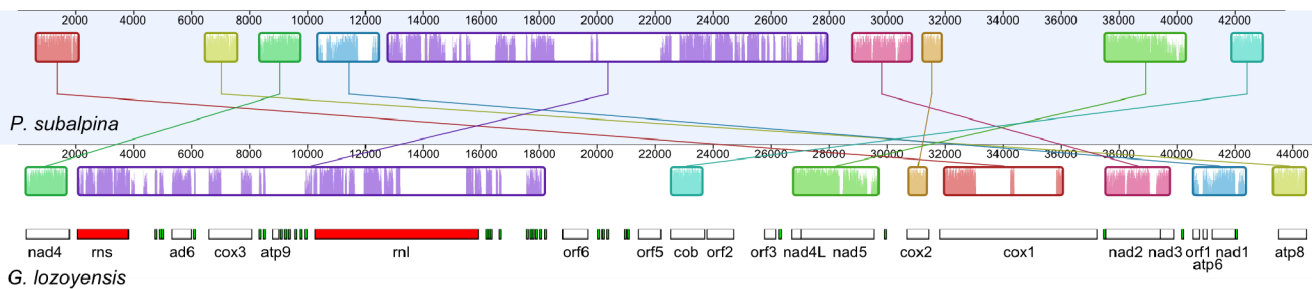**B**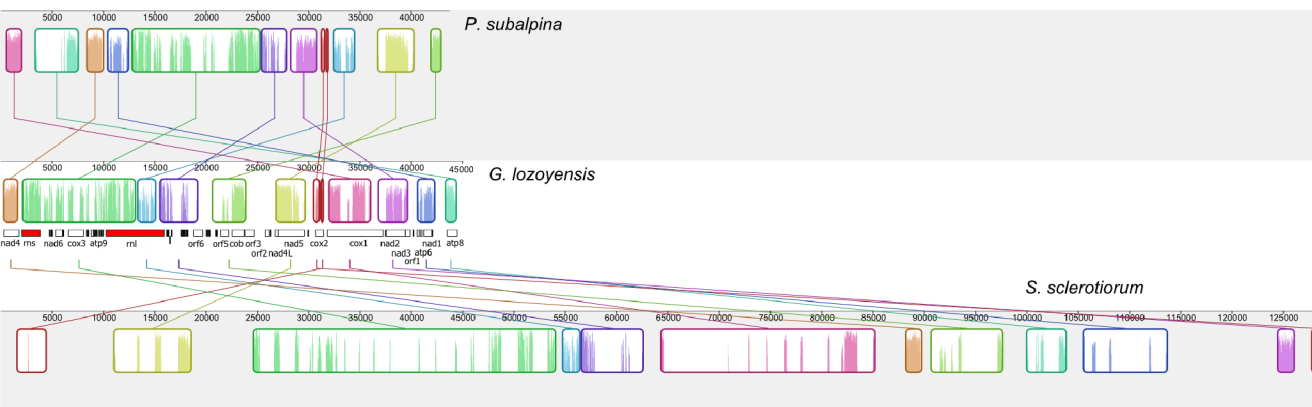

Supplement: Figure S2 — Mauve genome comparison. Multiple alignments of four helotialean species, G. lozoyensis, P. subalpina, S. sclerotiorum and B. cinerea [67] were performed with the Mauve software package [67]. Locally collinear blocks (LCB) of the genome sequences are shown in identical colors and are connected with lines. For the genome of G. lozoyensis the annotation is displayed to allow the assignment of genes to LCBs. a) Alignment of G. lozoyensis and P. subalpina. b) Alignment of G. lozoyensis, P. subalpina and S. sclerotiorum. (PDF) [file pone.0074792.s002.pdf]
